# Association between HIV PrEP indications and use in a national sexual network study of US men who have sex with men

**DOI:** 10.1002/jia2.25826

**Published:** 2021-10-03

**Authors:** Kevin M. Weiss, Pragati Prasad, Travis Sanchez, Steven M. Goodreau, Samuel M. Jenness

**Affiliations:** ^1^ Department of Epidemiology Emory University Atlanta GA USA; ^2^ Icahn School of Medicine at Mount Sinai New York NY USA; ^3^ Department of Anthropology University of Washington Seattle WA USA

**Keywords:** HIV, pre‐exposure prophylaxis, prevention, men who have sex with men

## Abstract

**Introduction:**

HIV pre‐exposure prophylaxis (PrEP) is effective in preventing HIV transmission. United States Public Health Service (USPHS) clinical practice guidelines define biobehavioral indications for initiation. To assess guideline implementation, it is critical to quantify PrEP nonusers who are indicated and PrEP users who are not indicated. We sought to estimate current PrEP use among US men who have sex with men (MSM), characterize whether their PrEP use aligned with their current indications for PrEP, and assess whether the association between PrEP indications and PrEP use differed by demography or geography.

**Methods:**

Using data from a US web‐based sexual network study of MSM between 2017 and 2019, we measured PrEP usage and assessed whether respondents met indications for PrEP. Log‐binomial regression was used to estimate the relationship between PrEP indications and PrEP use, with adjustment for geography, age and race/ethnicity.

**Results:**

Of 3508 sexually active, HIV‐negative MSM, 34% met indications for PrEP. The proportion with current PrEP use was 32% among MSM meeting indications and 11% among those without indications. Nearly 40% of those currently using PrEP did not meet indications for PrEP, and 68% of MSM with indications for PrEP were not currently using PrEP. After adjusting for geography and demographics, MSM with PrEP indications were about three times as likely to be currently using PrEP. This association varied slightly, but not significantly, by geographic region, age and race/ethnicity.

**Conclusions:**

Indications for PrEP strongly predicted current PrEP use among US MSM. However, we identified substantial misalignment between indications and use in both directions (indicated MSM who were not benefitting from PrEP, and MSM taking PrEP while not presently being indicated). PrEP underuse by those at greatest risk for HIV acquisition may limit the projected impact of PrEP implementation, despite reported increases in PrEP provision. This calls for further implementation efforts to improve PrEP delivery to those most in need during periods of elevated sexual risk and to close the gap between indications and uptake.

## 1 INTRODUCTION

Despite biomedical advances in human immunodeficiency virus (HIV) prevention with antiretroviral pre‐exposure prophylaxis (PrEP), the burden of HIV among men who have sex with men (MSM) remains high [1]. MSM are a high‐priority risk group for PrEP use based on their behavioral and biological risk factors [2, 3]. The United States Public Health Service (USPHS), a governmental public health agency, produces clinical guidelines for health care providers, most recently updated in 2017, which define specific biobehavioral indications for PrEP prescription for MSM [4].

PrEP is a key part of HIV strategy globally. In the United States, PrEP is available through multiple mechanisms including national and state health insurance programs covering health care costs for those with low income, private insurance plans, programs for those without health insurance and copay assistance from the drug manufacturer or US states [5, 6]. In 2019, the US Preventive Services Task Force, a body that reviews research and provides recommendations about evidence‐based clinical practices, gave PrEP the highest possible recommendation, which, under US law, prevents insurers from charging patients out‐of‐pocket costs for the medication or other associated clinic visits and laboratory tests [7, 8].

The population prevention impact of PrEP depends on coverage, or the proportion of indicated persons who use PrEP. Mathematical models and ecologic implementation data have estimated the association between PrEP coverage and lower HIV incidence [9] and diagnosis rates among MSM [10]. Data through the mid‐2010s indicated that only a small fraction of sexually active US MSM were estimated to be using PrEP [11, 12, 13], though uptake has increased over the past few years, with two recent estimates placing uptake among eligible MSM at 20% [14] and 35% [15]. Various factors, such as access to health care [16], likely contribute to sub‐optimal PrEP coverage. There is some evidence that patient‐perceived HIV risk may underestimate clinical assessments of HIV risk and eligibility for PrEP [17]. It is critical to characterize the group of MSM with indications for PrEP but who are not using it.

To achieve maximum prevention benefits under financial constraints, PrEP implementation efforts must also consider efficiency. Efficient delivery of PrEP means a low number needed to treat (NNT, quantified as person‐time on PrEP) to avert one new infection. Maximally efficient intervention targeting scenarios, which deliver interventions only to a group who would optimally benefit from it, have an NNT approaching 1. PrEP use in some groups of MSM who are at low likelihood of acquiring HIV (either through their own behavior or as a function of their epidemiological context) could substantially reduce PrEP efficiency while having a minimal impact on HIV incidence [9]. PrEP medication supplies are not currently limited and care rationing is unlikely, however use of PrEP clinical services by MSM not indicated for PrEP may also limit the potential efficacy of PrEP implementation.

In this study, we explore the two ‘off‐diagonal’ scenarios in the PrEP indication and uptake matrix: those who are indicated for PrEP but not using it, and those who are not indicated for PrEP but using it. Although the indications in the guidelines have imperfect sensitivity and specificity in capturing MSM who may be at risk for HIV acquisition, examining this misalignment may be the first step towards their re‐evaluation. It is particularly important to determine whether these associations differ among key MSM sub‐groups (geography, race/ethnicity, age) commonly studied in HIV prevention research to understand how misalignment may contribute to or correlate with differential HIV incidence and PrEP use.

Our primary research aims were to estimate the proportions of US MSM currently using PrEP compared to their indications, to quantify the strength of the association between indications and use and to better characterize whether any misalignment could be explained by or differ among sub‐groups. We hypothesized that PrEP indications would strongly predict PrEP use for US MSM, but that any misalignment between indications and use could vary by geography age, or race/ethnicity.

## 2 MATERIALS AND METHODS

### 2.1 Study design

We used data from the ARTnet study of cis‐gender US MSM for this analysis. The complete methods for ARTnet have been described previously [18, 19]. ARTnet was a web‐based sexual network study seeking to characterize sexual partnership networks among and engagement in HIV prevention services by MSM in the United States. Eligibility included any lifetime history of male‐male sex and age between 15 and 65 years. ARTnet participant data were linked to the participant's responses from the American Men's Internet Study (AMIS), a larger web‐based HIV behavioral survey [20] and then de‐duplicated. Study procedures were completed between 2017 and 2019. The Emory University Institutional Review Board approved the study protocol. The main study procedure was an online survey, which was hosted on a Health Insurance Portability and Accountability Act (HIPAA)‐compliant web platform (SurveyGizmo, Boulder, CO).

### 2.2 PrEP use

Two measures were used to calculate PrEP use. All participants reported a negative result on their last HIV test were provided with a short description of PrEP as an antiretroviral pill (Truvada), which could be taken every day to reduce a person's chance of getting HIV. Participant lifetime use of PrEP was then measured by the question ‘Have you ever taken PrEP (i.e., Truvada)?’ with participants who responded affirmatively also having current PrEP uptake assessed by the question: ‘Are you currently taking PrEP (i.e. Truvada)?’

### 2.3 PrEP indications and covariates

Survey data were used to evaluate whether participants reported behaviors that met indications under the updated clinical provider practice guidelines for PrEP prescription [4]. These measures included the number of recent sexual partners and additional individual‐ and partnership‐level information for up to the five most recent partners reported in the year prior to survey completion. These measures included: whether the partnership was with a main, casual, or one‐time partner, the dates of the partnership (start, end, most recent sexual activity, whether the participant thought that the relationship would continue), what sexual activities occurred with each partner (anal/oral intercourse, frequency of sexual acts sexual role and positioning), whether the participant and partner were using PrEP or anti‐retroviral therapy or condoms, and the respondent and partner's histories of a sexually transmitted infection (STI) diagnosis.

Two study sub‐populations were defined for the analysis. The first, referred to as *PrEP‐eligible MSM*, includes all MSM who reported being HIV‐negative and have been sexually active with another man in the prior 12 months. The subset of PrEP‐eligible MSM who met all indications for PrEP [4] was then defined, referred to hereafter as *PrEP‐indicated MSM*. To be categorized as indicated, participants had to: (1) report their most recent HIV test as negative; (2) not be in a monogamous partnership (per respondent definition) with an HIV‐negative partner; (3) report anal intercourse with another man in the prior 6 months; and (4) report either any condomless anal intercourse (CAI) with another man in the prior 6 months or a diagnosis of gonorrhea, chlamydia or syphilis in the prior 6 months. The guidelines for PrEP indications also include being aged 18 or older, but we excluded that in this analysis to better evaluate eligibility based on behavior among 15‐ to17‐year‐old respondents.

Participant‐reported survey data were collected for other covariates. Participant‐reported ZIP codes were matched to one of four census regions (South, Midwest, Northeast West) [21], while reported race/ethnicity was grouped into four categories: Non‐Hispanic Black, Non‐Hispanic White, Hispanic, Other race/ethnicity. Additionally, reported ages were grouped into categories: 15–17; 18–24; 25–34; 35–44; 45–54 and 55–65.

### 2.4 Statistical analysis

For our exploratory analysis, we present descriptive analyses of persons indicated for and using PrEP by demographic category using proportions and standard deviations. To quantify the association between PrEP indications and use, we selected a primary exposure of having USPHS indications for PrEP with a primary outcome of current PrEP use. We used log‐binomial regression models to quantify this association using prevalence ratios (PR) and 95% confidence intervals (CI). After calculating the crude association, we estimated the multivariable PRs after adjustment for race/ethnicity, age category and geography. We also evaluated how the association between PrEP indications and current PrEP use might differ when stratified by demographic categories. All data analysis was performed in *R* 3.4 [22].

## 3 RESULTS

Of the 3508 PrEP‐eligible men included in the analysis, 631 (18%) reported currently using PrEP and 2877 (82%) reported not currently using PrEP. Table 1 summarizes current PrEP use by the presence of indications for its use. More than one‐third (34.0%) met indications for PrEP. More than one‐half (61.0%) of those currently taking PrEP met indications for PrEP, while 39.0% of current PrEP users did not. One‐third (n = 385, 32.2%) of PrEP‐indicated MSM reported currently using PrEP. Of the 2314 MSM who did not meet indications for PrEP, 246 (10.6%) were currently using PrEP. In total, nearly one‐third (30.1%) of the 3508 PrEP‐eligible MSM had PrEP usage misaligned with their current indications: 7.0% (n = 246) of PrEP‐eligible MSM were currently using PrEP despite not meeting indications for PrEP and 23.1% (n = 809) of PrEP‐eligible MSM were not using PrEP despite meeting indications for PrEP.

**Table 1:** Indications for PrEP and Current and Ever PrEP Use among PrEP‐Eligible MSM

	Current PrEP Use	Not Currently Using PrEP	Total
Meets Indications for PrEP** (Col%)	385 (61.0)	809 (28.1)	1194 (34.0)
Does Not Meet Indications for PrEP (Col%)	246 (39.0)	2068 (71.9)	2314 (66.0)
Total (Row%)	631	2877	3508

* PrEP‐Eligible: (1) HIV‐negative; and (2) Sexually active with a man in the past 12 months.

** Meet all US Public Health Service indications for PrEP (Base + either recent condomless anal intercourse or recent STI)—*note*: *excludes 18+ indication for those aged 15–17 (n = 25)*.

Table 2 summarizes current PrEP usage by demographic characteristics and PrEP indication status. Geographically, current PrEP use among indicated MSM differed significantly by geography, with use lowest in the South (28.7%) and greatest in the West (38.4%). PrEP use increased with age, with lowest use among 15–17 (16.7%) and 18‐ to 24‐year‐old MSM (14.1%) and increasing use in age groups greater than 24 years, culminating in highest PrEP use (38.1%) among 45‐ to 54‐year‐old MSM. Among MSM meeting indications for PrEP, there was no significant variation in current PrEP use by race/ethnicity, with use lowest among Hispanic MSM (30.2%). Among MSM not meeting indications for PrEP, PrEP usage was greatest among non‐Hispanic White (11.1%), Northeast (11.7%) and 45‐ to 54‐year‐old (14.9%) MSM, though these proportions differed significantly only by age category.

**Table 2:** PrEP use by indication status and demographics among PrEP‐Eligible MSM*

	Meeting indications for PrEP**			Not meeting indications for PrEP			
	Total	Current PrEP use	Not using PrEP	Total	Current PrEP use	Not using PrEP	Current PrEP users Meet
	N	N (Row%)	N (Row%)	N	N (Row%)	N (Row%)	indications %
**Overall**	1194	385 (32.2)	809 (67.8)	2314	246 (10.6)	2068 (89.4)	61.0
***US Census Region***	*p =* 0.04			*p = 0.55*			*p =* 0.55
Northeast	214	71 (33.2)	143 (66.8)	436	51 (11.7)	385 (88.3)	58.2
Midwest	252	75 (29.8)	177 (70.2)	440	51 (11.6)	389 (88.4)	59.5
South	418	120 (28.7)	298 (71.3)	853	81 (9.5)	772 (90.5)	59.7
West	310	119 (38.4)	191 (61.6)	585	63 (10.8)	522 (89.2)	65.4
***Age Category***	*p* < 0.01			*p* < 0.01			*p* < 0.01
15–17**	6	1 (16.7)	5 (83.3)	19	0 (0.0)	19 (100.0)	100.0
18–24	213	30 (14.1)	183 (85.9)	551	40 (7.3)	511 (92.7)	42.9
25–34	331	118 (35.6)	213 (64.4)	693	81 (11.7)	612 (88.3)	59.3
35–44	205	76 (37.1)	129 (62.9)	342	44 (12.9)	298 (87.1)	63.3
45–54	252	96 (38.1)	156 (61.9)	349	52 (14.9)	297 (85.1)	64.9
55–65	187	64 (34.2)	123 (65.8)	360	29 (8.1)	331 (91.9)	68.8
***Race/Ethnicity***	*p* = 0.95			*p* = 0.68			*p* = 0.80
Non‐Hispanic White	871	284 (32.6)	587 (67.4)	1705	189 (11.1)	1516 (88.9)	60.0
Non‐Hispanic Black	49	16 (32.7)	33 (67.3)	104	10 (9.6)	94 (90.4)	61.5
Hispanic	172	52 (30.2)	120 (69.8)	302	27 (8.9)	275 (91.1)	65.8
Other	102	33 (32.4)	69 (67.6)	203	20 (9.9)	183 (90.1)	62.3

* PrEP‐Eligible: (1) HIV‐negative; and (2) Sexually active with a man in the past 12 months.

** Meet all US Public Health Service indications for PrEP (Base + either recent condomless anal intercourse or recent STI)—*note: excludes 18+ indication for those aged 15–17 (n = 25)*. ** Meet all USPHS indications for PrEP (Base + either recent condomless anal intercourse or recent STI)*—note*: *excludes 18+ indication for those aged 15–17 (n = 25)*.

*p*‐values are for Chi‐Square test.

Table 2 also includes the proportion of current PrEP users meeting PrEP indications by category. The proportion meeting PrEP indications varied little by race/ethnicity, ranging from 60.0% among non‐Hispanic White MSM to 65.8% of Hispanic MSM. PrEP‐using MSM in the West (65.4%) were the only geographic sub‐group where the percentage meeting indications for PrEP exceeded the overall average (61.0%). Younger PrEP‐using MSM were less likely to meet indications for PrEP, apart from the small 15‐ to 17‐year‐old cohort, with proportions increasing significantly by age.

In crude regression analyses, MSM who met indications for PrEP were 3.03 (CI: 2.63, 3.51) times as likely as those not meeting indications to be currently taking PrEP (Table 3). In crude analyses, only MSM in the West were significantly more likely than Southern MSM to be using PrEP (PR = 1.29, CI: 1.07, 1.54). The likelihood of current PrEP use increased 1.12 times (CI: 1.07, 1.18) with a 10‐year increase in age. The relative prevalence of current PrEP use among non‐White participants was marginally lower (from 5 to 9% lower) than White participants in crude analyses but these estimates did not differ significantly. The association between PrEP indications and PrEP use was not explained by the covariates, changing little (PR = 2.98) after adjustment for geography, age and race/ethnicity.

**Table 3:** Crude and adjusted log‐binomial regression correlates of current PrEP use among PrEP‐eligible MSM

Correlate	Crude prevalence ratio (95% CI)	Adjusted prevalence ratio (95% CI)
***PrEP Indications***		
Meet Indications for PrEP	3.03 (2.63, 3.51)	2.98 (2.59, 3.45)
***Census Region***		
Northeast	1.19 (0.97, 1.45)	1.18 (0.97, 1.43)
Midwest	1.15 (0.94, 1.41)	1.10 (0.90, 1.33)
South	*—*	*—*
West	1.29 (1.07, 1.54)	1.30 (1.09, 1.55)
***Age***		
Age (10‐year increase)	1.12 (1.07, 1.18)	1.10 (1.04, 1.15)
***Race/Ethnicity***		
Non‐Hispanic White	*—*	*—*
Non‐Hispanic Black	0.93 (0.63, 1.29)	1.02 (0.70, 1.40)
Hispanic	0.91 (0.72, 1.12)	0.93 (0.97, 1.15)
Other	0.95 (0.72, 1.21)	0.98 (0.75, 1.24)
CI: Confidence Interval		

When evaluating how stratified analysis might affect the association between PrEP indications and PrEP use, we found that the regression point estimates for the magnitude of association varied when limited to specific sub‐groups, but the CIs were overlapping (Table 4). The estimate of the association was greatest among MSM in the West (PR: 3.56) and weakest in the Midwest (PR: 2.57). When evaluating the relationship between PrEP indications and PrEP use among racial/ethnic participant sub‐groups, the magnitude of association was greatest among non‐Hispanic Black (PR: 3.40) and Hispanic (PR: 3.38) participants and weakest among White participants (PR: 2.94).

**Table 4:** Crude log‐binomial regression prevalence ratios for the association between PrEP indications and PrEP use in sub‐group‐specific models among PrEP‐eligible MSM

Correlate	Crude prevalence ratio (95% CI)
***Census Region***	
Northeast	2.84 (2.07, 3.93)
Midwest	2.57 (1.87, 3.56)
South	3.02 (2.35, 3.92)
West	3.56 (2.73, 4.71)
***Race/Ethnicity***	
Non‐Hispanic White	2.94 (2.50, 3.47)
Non‐Hispanic Black	3.40 (1.69, 7.24)
Hispanic	3.38 (2.23, 5.26)
Other	3.28 (2.01, 5.54)

* All prevalence ratios represent the crude log‐binomial regression estimates for the association between PrEP indications and current PrEP use (Crude Ratio for entire study population: 3.03 (2.63, 3.51)).

## 4 DISCUSSION

In this study, we found evidence of substantial misalignment between the US Public Health Service indications for PrEP and current PrEP use among PrEP‐eligible MSM. Having indications for PrEP strongly predicted PrEP use, but nearly one‐third of PrEP‐eligible MSM had possible misalignment between indications and use. The misalignment was greater for MSM indicated for PrEP but not using it (underuse) compared to MSM not indicated for PrEP but using it. Overall, 68% of PrEP‐indicated MSM (23% of all PrEP‐eligible MSM) were not using PrEP, and 39% of MSM currently using PrEP (7% of all PrEP‐eligible MSM) were not indicated for PrEP. This suggests overall that PrEP underuse remains a public health priority in the United States, with continued efforts needed to close the gap between indications and uptake.

Across all demographic and geographic sub‐groups, there was a sizeable pool of PrEP‐eligible US MSM who met indications for, but were not currently taking, PrEP. Control of the HIV epidemic with PrEP depends on a relatively high level of coverage [9, 10], but persistent PrEP underuse among at‐risk MSM could limit the possibility of meeting national and local HIV prevention goals in the United States [5] or in any other setting. Our finding, that a significant number of MSM meeting biobehavioral indications, indicating increased risk for HIV acquisition, were not using PrEP aligns with previous work focusing on the interaction of eligibility and use [23]. Using the framework of a PrEP care continuum [24, 25], many individual‐ and structural‐level barriers may limit otherwise suitable candidates from benefitting from using the medication. These include patient barriers such as decreased HIV risk perception [17] as well as provider barriers (lack of knowledge, stigma, and concerns about cost, behavior or adherence) described in a recent review [26]. These barriers, however, highlight potential points of interventions to improve access, use, adherence and persistence on PrEP [12, 27, 28].

A large percentage (38%) of MSM in this study who currently used PrEP did not presently report indications for its use. Previous modeling has shown that the efficiency of PrEP (e.g. number needed to treat) for both HIV and associated prevention (STI screening) depends on the target population, with decreased efficiency, and efficacy in some cases, when PrEP is provided to individuals who are at lower risk [9, 29]. Theoretically, though never observed, use of PrEP by those presently without behavioral indications, or a ‘worried well’ population, requires societal resources (in terms of public and private funding of medications via health care insurance payments) and use of clinical services that may not generate as large of a clinical benefit. Though social desirability bias could play a role in potential under‐reporting of HIV status or risk behaviors, the 1 in 10 non‐indicated MSM who were using PrEP represent a potential intervention target for further patient or provider education that reflects the dynamic nature of behavior and indications for PrEP. To the hypothesis that provider and patient knowledge of PrEP and HIV risk can be improved, the guidelines for PrEP indications provide a benchmark for how PrEP determination can be assessed [30]. An informed discussion with a provider about PrEP being one tool among other proven risk reduction strategies, such as on‐demand PrEP (2:1:1) [31, 32] or PEP or condom usage, could be important for MSM not currently meeting indications, but this relies on ensuring trust between patients and providers to obtain an accurate sexual history [28]. As global PrEP scale‐up continues, it is essential that resources be focused to those who may benefit the most from PrEP, including associated services such as counseling and regular STI screening, and may face the greatest barriers to uptake.

Few other assessments are able to concurrently present PrEP indications and eligibility alongside PrEP use [14, 15, 33]. Current PrEP uptake estimates among eligible MSM in this study (32.2%) tracked closely with recent estimates of PrEP uptake the prior year among US MSM likely meeting PrEP indications (HIV‐negative with either a HIV‐positive partner or multiple male partners and either CAI or a recent STI) in major metropolitan areas surveyed through National HIV Behavioral Surveillance (NHBS) [15]. These estimates among indicated MSM exceed reports of prior‐year PrEP use among surveyed US MSM in the American Men's Internet Survey (19.9%) and NHBS (25.0%) [14, 33]. Although these studies may differ in mode of data collection (online vs. in‐person) as well as different sampling frames (e.g. urban MSM), these findings, in addition to the ubiquity of internet access among US residents [34] and the experiences of large internet‐based surveys of sexual behaviors among MSM [35], support the use of web‐based studies in providing feasible, cost‐effective estimates of PrEP indications and uptake among MSM to complement in‐person or database‐driven estimates. Globally, PrEP initiation and uptake has been greatest in locations where it was adopted early, supported nationally and provided alongside other key services [38]. These estimates are specific to the evaluation of the USPHS guidelines in the United States and may differ from international or country‐specific PrEP eligibility guidelines in other countries that may differ in sensitivity or specificity by measuring risk in different ways [36, 37]. Web‐based studies or other assessments can help to assess the applicability of these findings in other settings.

Given the observed gaps in both HIV incidence and PrEP use among MSM of color, particularly black and Hispanic MSM, assessing the association between PrEP indications and PrEP use matters for work toward disparity reduction. Previous research has demonstrated that an equal or greater proportion of people of color in the United States, compared to White people, meet indications for PrEP [11], but make up only a small fraction of PrEP prescriptions [39]. MSM of color have seen HIV diagnosis rates remain stable or increase while rates decreased among White MSM [1], have lower comparative levels of PrEP use [33], and, in some studies, persons of color were less likely to be indicated for or receive PrEP [40]. In our analysis, the magnitude of association between PrEP indications and current PrEP use was greatest when limited to non‐Hispanic Black or Hispanic participants, possibly indicating lesser misalignment (or greater efficiency) between the guidelines and use in these groups. The significance of this finding deserves further investigation, as it does not necessarily align with studies that have found difficulty translating interest into uptake [41] and a lagged awareness of PrEP and HIV prevention in these same populations [42]. Multiple explanations likely contribute to this context, including potential individual (behavioral, psychological, risk perception, stigma, medical mistrust, self‐efficacy) [17, 27, 43] and structural (insurance, cost, access to care, health utilization) barriers to accessing and using PrEP for Black and other MSM of color that may reduce uptake and lead to decreased sensitivity of the guidelines in assessing HIV acquisition risk [44].

Given the high HIV burden and challenges with accessing healthcare in the US South, we hypothesized that PrEP use there would be lower than in other regions. The highest rates of HIV diagnosis are in the US South [1], as well as the lowest levels of PrEP use [45, 46, 47]. PrEP clinics are unevenly distributed across the United States [48], and the current number of clinics in the South may not be able to support the estimated need for PrEP [49]. In this study, PrEP‐eligible and PrEP‐indicated MSM in the US South reported the lowest levels of current PrEP usage, though the pattern was not dissimilar from other regions. As a crude measure of regional differences, the magnitude of the association between PrEP indications and use varied more geographically than when stratified by other characteristics. These crude measures may be influenced by other variables (possibly race/ethnicity), and likely need further exploration to determine their importance and whether they further highlight additional barriers to starting PrEP, particularly given region‐specific structural and individual barriers to PrEP uptake. A recent review of PrEP implementation strategies in the South highlighted a number of individual and structural factors, including a greater rural population, lesser access to PrEP care, fewer insured individuals, lower health literacy and HIV risk perception, and greater anti‐HIV, anti‐gay, and PrEP stigma, that likely influence PrEP uptake in the South [50]. Barriers to PrEP are not unique to the South; the estimated association between indications and use was weakest in the Midwest, where many of the same factors, such as stigma and limited access to PrEP care and information [51, 52], and some additional ones, such as rurality [51], may limit potential PrEP uptake. Novel implementation strategies, such as telemedicine‐based PrEP to better reach people in rural areas in the US [53, 54] or consideration of networks and social capital [55, 56], may be necessary to try to offset the barriers observed for PrEP uptake in the South, Midwest and indeed everywhere in the US.

### 4.1 Limitations

This analysis has some limitations. These data represent a convenience sample of MSM recruited online from across the United States and are not representative of all MSM including potential under‐representation of racial and ethnic minority MSM [57]. Ideally, these results could be interpreted in the context of other representative samples of US MSM, but none exist. However, we conducted stratified analysis by three core factors (race, age geography) in which there may be imbalances to better estimate group‐specific outcomes. With the assumption of conditional exchangeability, this partially alleviates this issue. In any online study, such as ARTnet, social desirability bias or reluctance to disclose personal information may be a factor, potentially resulting in overestimation of desirable prevention behaviors such as PrEP uptake or under‐reporting of HIV status or risk behaviors (and thus PrEP‐eligible MSM). Our use of the most recent HIV test result to determine HIV status could underestimate the proportion of MSM with indications for PrEP by undercounting MSM who are truly at‐risk (sexually active) but have not recently tested or ever have been tested. Finally, these data were limited to an ‘Other’ race/ethnicity category, but further analyses should ensure that race/ethnicity is explored in a more nuanced way.

## 5 CONCLUSIONS

Routine monitoring of PrEP uptake is needed to measure progress toward and gaps in PrEP coverage. Current PrEP uptake among US MSM tracked with prior estimates and meeting PrEP indications was strongly associated with current PrEP usage. However, there are populations of MSM who are indicated for but not using PrEP, as well as MSM who are using PrEP while not currently meeting the indications. The relative importance of behavioral indications and demographic differences in PrEP uptake highlight potential barriers to and gaps in implementing PrEP, both in the United States and globally, which will need to be addressed to meet PrEP's full potential to reduce new infections, particularly given efforts to both scale‐up and address structural barriers to accessing PrEP in the United States.

## COMPETING OF INTERESTS

The authors certify that they have NO affiliations with or involvement in any organization or entity with any financial interest (such as honoraria; educational grants; participation in speakers’ bureaus; membership, employment, consultancies, stock ownership, or other equity interest; and expert testimony or patent‐licensing arrangements), or non‐financial interest (such as personal or professional relationships, affiliations, knowledge or beliefs) in the subject matter or materials discussed in this manuscript.

## AUTHORS’ CONTRIBUTIONS

KMW and SMJ developed and executed the study, conducted the analyses and wrote the manuscript. SMG provided input on the study design, provided critical input on the analysis and critically reviewed and edited the manuscript. PP and TS provided input on the study design and critically reviewed and edited the manuscript.

## FUNDING

This work was supported by National Institutes of Health Grants R21 MH112449 and a Grant from the MAC AIDS Fund.

## Data Availability

Data are available from the senior author upon request.
